# Supplementary material for: Investigating the influence of physiologically relevant hydrostatic pressure on CHO cell batch culture
Source: Sci Rep. 2021 Jan 8;11:162. doi: 10.1038/s41598-020-80576-8 (PMC7794228; doi:10.1038/s41598-020-80576-8)
Supplement: Supplementary file 1 — Supplementary Information 1. [file 41598_2020_80576_MOESM1_ESM.zip › S1/IgG/Jan17 standard curve/40ugml.pdf]

Data File C:\Users\P...s\ChemStation\1\Data\TK_CHO_IgG 2019-01-17 13-50-44\003-3-40ug_ml.D
Sample Name: 40ug/ml

===

Acq. Operator : SYSTEM Seq. Line : 3
Sample Operator : SYSTEM
Acq. Instrument : HPLC-0039 Location : 3
Injection Date : 1/17/2019 2:00:52 PM Inj : 1
Inj Volume : 100.000 µl
Acq. Method : C:\Users\Public\Documents\ChemStation\1\Data\TK_CHO_IgG 2019-01-17 13-50-44\CHO_IgG_LC.M
Last changed : 12/4/2018 1:38:41 PM by SYSTEM
Analysis Method : C:\Users\Public\Documents\ChemStation\1\Data\TK_CHO_IgG 2019-01-17 13-50-44\CHO_IgG_LC.M (Sequence Method)
Last changed : 1/17/2019 2:21:41 PM by SYSTEM
(modified after loading) (Current integration events modified)

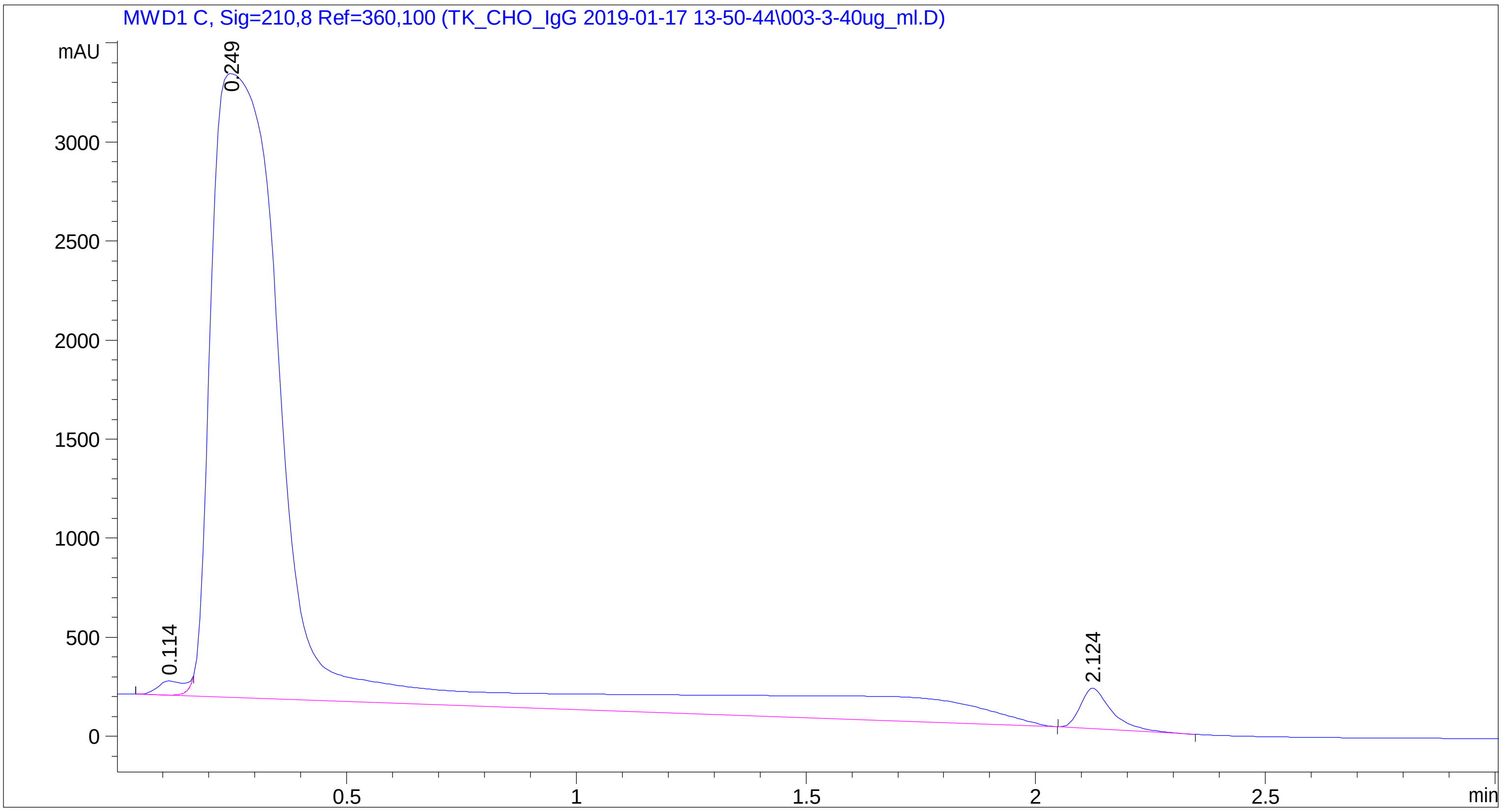

===

Area Percent Report

===

Sorted By : Signal
Multiplier : 1.0000
Dilution : 1.0000
Use Multiplier & Dilution Factor with ISTDs

Signal 1: MWD1 C, Sig=210,8 Ref=360,100

Peak #	RetTime [min]	Type	Width [min]	Area [mAU*s]	Height [mAU]	Area %
1	0.114	BV E	0.0546	276.51413	72.28306	0.6860
2	0.249	VB R	0.1908	3.90631e4	3147.21606	96.9164
3	2.124	BBA	0.0711	966.34979	204.64519	2.3975

Totals : 4.03060e4 3424.14431

===

*** End of Report ***
